# Assessing Work-Related Stressors in Online Counseling: Cross-Sectional Questionnaire Development Study

**DOI:** 10.2196/65766

**Published:** 2025-09-25

**Authors:** Wiebke Schlenger, Marlies Joellenbeck, Elke Ochsmann

**Affiliations:** 1 Institute of Occupational Health, Prevention and Health Management, Faculty of Medicine, Lübeck University, Luebeck, Germany; 2 Institute of Occupational Medicine and Public Health, Faculty of Medicine, Saarland University, Kirrberger Str. 100, Homburg/Saar, 66424, Germany, 49 68130275030

**Keywords:** workplace risk assessments, digital transformation, questionnaire, online counseling, therapeutic relationship, work organization, employee health

## Abstract

**Background:**

The rapid digitalization of health and social services, particularly accelerated by the COVID-19 pandemic, has led to a widespread adoption of online counseling. While offering flexibility and improved access for clients, online counseling presents new challenges for counselors, including technical issues, difficulties in building therapeutic relationships, and changes in work organization. Despite growing reliance on digital counseling platforms, there is a lack of validated tools to assess specific occupational stressors associated with online counseling.

**Objective:**

This study aimed to develop and evaluate the “QueStrOn” (Questionnaire to Assess Stressors in Online Counseling), an instrument designed to identify stressors and resources specific to online counseling and to explore its ability to predict perceived stress levels in counselors.

**Methods:**

Item development was guided by the Job Demands–Resources model, qualitative interviews with 22 counselors, expert input, and a literature review. A preliminary version of the questionnaire was pretested and then distributed via email and social media to counselors offering both online and face-to-face services. A total of 219 counselors completed the survey, and after applying inclusion criteria, 174 responses were analyzed. Exploratory factor analysis was conducted using principal axis factoring and varimax rotation. Internal consistency was assessed via Cronbach alpha (α). A linear regression model was used to initially test the predictive power of the identified factors with perceived digital stress as the dependent variable.

**Results:**

The exploratory factor analysis resulted in a four-factor solution with 16 items, capturing (1) Online Work Organization, (2) Online Framework, (3) Online Work Content, and (4) Online Communication. The overall instrument demonstrated high internal consistency (α=0.870), with acceptable values for factors (1), (3), and (4) (α=0.754, 0.745, and 0.826, respectively), although the factor “Online Framework” showed limited reliability (α=0.502). The regression model, adjusted for age and gender, significantly predicted perceived stress in online counseling (*F*_5_=13.335, *P*<.001), explaining 27.1% of the variance. Online Work Organization, Online Communication, and Online Framework were associated with lower perceived stress when rated positively, whereas Online Work Content showed an inverse relationship, potentially reflecting emotional distancing.

**Conclusions:**

The QueStrOn instrument provides a valid first step toward systematically assessing occupational stressors in online counseling. Its 4-factor structure aligns with theoretical and empirical findings and offers practical utility for workplace risk assessments. Incorporating these dimensions into routine evaluations may support counselor well-being and inform digital health policy. Further validation and longitudinal studies are recommended to expand its applicability and explore associations with broader health outcomes.

## Introduction

### Background

While face-to-face counseling has been established for decades, the use of online counseling has been increasing since the year 2000 [1], with a sharp rise during the COVID-19 pandemic [2-4], when the demand for counseling appointments skyrocketed amidst social isolation caused by lockdown measures. A survey by the American Psychiatric Association found that many counselors (81%) now use online counseling significantly more frequently than before the pandemic [4]. Similarly, a representative survey in Germany reported that approximately 50% of counseling institutions now use video counseling with their clients, compared to just 5% before the pandemic [3].

### Occupational Challenges in Online Counseling

Counselors report a high workload, frequent overtime, and emotional strain resulting from the complex and emotionally affecting issues presented by their clients [5,6]. These occupational risk factors are known to increase the prevalence of personal distress among counselors [7,8]. Personal distress can negatively affect health, reduce job satisfaction, and decrease willingness to use information and communication technology in the workplace [7,9,10].

The increased use of online counseling may introduce additional stressors for counselors, highlighting the need for tailored occupational health assessments and recommendations. Previous research indicated that many online counselors experience difficulties with the digital mode of counseling [11,12], as it may challenge the therapeutic relationship due to changes in counseling settings and altered facial expressions [12-15]. Both factors have been reported to affect the therapeutic relationship [14,16,17]. Asynchronous online counseling is particularly challenging for conveying emotions and maintaining clear communication [12,15,18]. Also, counseling techniques effective in face-to-face sessions, such as role plays or spatial experiments, may be less effective or insufficient in digital settings [15,19]. Achieving counseling objectives, such as establishing a therapeutic relationship and experiencing meaningful work, significantly contributes to job satisfaction [11,20,21].

Additional challenges of online counseling include variability in technology availability and proficiency, as well as client anonymity [12,22,23]. Technical difficulties can delay responses and disrupt counseling progress [24]. A stable internet connection and adequate technical equipment are necessary to experience the benefits of online counseling [25,26].

Client anonymity has the potential to facilitate disclosure by increasing clients’ sense of safety, but it can also complicate assessments of client intentions and crisis interventions [18,23,27]. The increased use of online counseling may therefore negatively impact the therapeutic relationship and counseling outcomes compared to face-to-face counseling [14,17,28]. While some studies show that clients rate the therapeutic relationship of online counseling as comparable or even superior to that of face-to-face counseling, counselors and therapists still express concerns regarding the efficacy of online counseling and therapy [2,17,28,29].

### Occupational Health Measures

Digitalization has been identified as a major driver for recent developments in the world of work [30-32]. While it may offer advantages and the potential to improve work performance and job satisfaction, it can also contribute to the development of distress and associated health risks [33,34]. Uncertainties related to technology use, social isolation from working in decentralized teams, and rapid information gathering and processing resulting from technology overload can lead to personal distress, burnout, and depression [7,34,35]. Occupational health and safety standards for online counseling, aimed at mitigating the development of psychological problems in the workplace, may become necessary [36,37]. In Germany, the German Occupational Health and Safety Act mandates systematic risk assessments and implementation of corresponding preventive measures [36].

### Research Question

To our knowledge, no validated quantitative survey instruments currently exist for assessing specific stressors experienced by online counselors. This study aims to develop a framework for a questionnaire (“QueStrOn” [Questionnaire to Assess Stressors in Online Counseling]) designed to evaluate specific health risks associated with this kind of work. In addition, the interplay between questionnaire results and distress of online counselors was explored.

## Methods

### Study Design and Participants

For participant recruitment (invited convenience sample), counselor contact lists of the former BZgA (Bundeszentrale für gesundheitliche Aufklärung, Federal Center for Health Education, now: BIÖG: Federal Institute of Public Health) were reviewed, and counselors or counseling centers qualified for the study (ie, those offering online counseling via personal contact methods, such as email or video) were contacted by email [38]. A total of 844 counselors or counseling centers qualified and were invited to participate. However, the final number of counselors working within these centers could not be determined using this approach.

The study was also promoted in selected social media groups where counselors and psychotherapists offer online counseling services. Potential participants received an email with detailed information about the study’s purpose, procedures, and estimated survey duration, along with a link to participate. Additional contact information was provided for any follow-up questions. The study information was also posted on the institute’s home page.

The cross-sectional survey was conducted from July to September 2021 on the platform SoSci Survey [39]. Participation was voluntary. Altogether, 219 questionnaires were completed in full (completeness was checked after questionnaire submission). Inclusion criteria for analysis required participants to conduct both online and face-to-face consultations. Thus, 174 questionnaires met these criteria and were included in the analysis. The overall completion rate was 219 out of 361 (ie, the ratio of users who finished the survey to those who initially agreed to participate).

### Questionnaire Development

The construction of the questionnaire to assess the stressors and resources of online counselors was based on the theoretical model of Job Demands and Resources by Bakker and Demerouti [40].

Specific stressors related to online counseling were initially identified through a qualitative study involving 22 consultants from a large German welfare organization, as described elsewhere [11]. The responses indicated that digitization influences all known areas of work-related stressors with overlaps between digital and nondigital work, including counseling activity design, emotional involvement, working hours, communication, work environment, and social relations (colleagues, supervisors, and flexible working conditions). However, counselors also distinguished between digital and nondigital work, suggesting that stressors specific to online counseling, and thus relevant to digital occupational health, warrant further investigation.

These findings were then compared and validated by a comprehensive literature review focusing on factors of the common guideline for psychosocial risk assessments in Germany (GDA [Gemeinsame Deutsche Arbeitsschutzstrategie]; Joint German Occupational Safety and Health Strategy guideline for mental stress) [41]. Challenges related to work organization and limitations in establishing therapeutic relationships were identified as specific stressors in online counseling, whereas other areas (eg, social relations) were not considered unique for digital or online work. Work organization was further defined by clients’ spatial and temporal flexibility, accompanied by changes in time management and documentation. Therapeutic relationships encompassed aspects like counselor-patient communication and nonverbal interaction.

The resulting questionnaire comprising 19 items was developed according to the established work organization and therapeutic relationships categories. These items were all placed on 1 questionnaire page. Sociodemographic data and health data were presented on other questionnaire pages. Responses consisted of paired negative and positive statements, rated on a Likert scale ranging from –3 (the aspect is a disadvantage of online counseling) to +3 (the aspect is an advantage of online counseling). Respondents were allowed to review and modify their answers.

In addition to exposure assessments, a stress outcome variable was included, asking participants to rate their stress level during online counseling compared to face-to-face counseling on a scale from (–3) “more stress during online counseling” to (+3) “more stress during face-to-face counseling.”

A questionnaire for psychosomatic complaints in nonclinical settings was also administered [42].

The final questionnaire was refined following consultations with a psychologist, a health scientist, and an occupational physician, all with experience in mental health risk assessments. It was pretested online with 2 online counselors and technically checked for desktops (the use of mobile phones for participation was not recommended). The questionnaire items for the QueStrOn had to be filled in (at least by checking a box for “no answer”) and participants were able to review and alter their answers before submission (by using a back button).

### Statistics

Following descriptive analyses, an exploratory factor analysis (EFA) was conducted (principal axis method, varimax rotation, and eigenvalue >1). To assess the suitability of the variables for the EFA, the KMO (Kaiser-Meyer-Olkin) criterion (KMO>0.8) as well as the Bartlett Sphere Test (*P*<.05) were applied. Factor selection was also guided by interpretability and content relevance. Cronbach alpha was calculated to evaluate the reliability of the scales.

The outcome variable “digital stress” was further examined for gender and age effects using the chi-square test. Its correlation with psychosomatic health outcomes (back pain, nervousness, and sweat attacks) was assessed. The predictive power of the questionnaire for digital stress was evaluated using linear regression analysis with standardized beta coefficients and T-values reported. A significance level of *P*<.05 was considered statistically significant. Statistical analysis was performed using SPSS 28.0 (IBM Corp [43]).

### Ethical Considerations

The study was conducted in accordance with the Declaration of Helsinki and approved by the Institutional Ethics Committee of the University of Lübeck (protocol code AZ 19‐353, approval date November 13, 2019). Participation was entirely voluntary, and no technical backtracking of respondents was possible. Respondents provided informed consent by reading detailed online information about the study and actively agreeing to complete the questionnaire before participation.

All data were collected and processed anonymously to protect participants’ privacy, and this was clearly communicated to them. Measures were taken to ensure confidentiality, including secure data storage and restricted data access to study personnel only. Personal information was chosen, ensuring that participants could not be identified in any reports or publications. Participants were not compensated for their involvement in the study.

## Results

### Participant Characteristics

A total of 78% (136/174) of respondents were female. The mean age of participants was 46.6 (SD 11.3; range 24-72) years. Altogether, 39.6% (69/174) of respondents were employed full-time, 51.7% (90/174) were employed part-time, and 20.1% (35/174) were self-employed or freelancers.

Psychotherapists (46/174, 26.4%) comprised the largest occupational group, while the remaining respondents worked in various fields of social counseling. Video counseling was the most frequently used format for online counseling (132/174, 75.8% of participants). Nevertheless, face-to-face counseling still accounted for the majority of participants’ working time.

### Online Counseling Stressors and Resources

Regarding work organization, clients’ spatial and temporal flexibility was perceived as an advantage of online counseling. Time for documenting digital or online sessions was reported to be similar to that of face-to-face counseling, and only minimal changes in counselors’ time management were documented. However, 68.4% (119/174) of counselors stated that technical difficulties interfered with the counseling process.

Aspects supporting a strong therapeutic relationship were rated as more negative in online counseling compared to face-to-face sessions. Specifically, 71.8 % (125/174) of the respondents reported that altered facial expressions and gestures negatively influence the therapeutic relationship, and 66.1% (115/174) of the respondents found the process of relationship building to be generally more difficult online. In addition, 77.2% (134/174) of the respondents stated that crisis management was more challenging in online counseling. While 49.5% (86/174) of counselors felt that they can express themselves clearly in online counseling, only 38.1% (66/174) believed that clients can do so.

### Online Stress

With regard to stress outcomes, 34.1% (59/173) of counselors reported higher stress levels during online counseling compared to face-to-face sessions. Approximately 37% (64/173) of counselors experienced no difference in stress between the 2 counseling forms, and 29.5% (51/173) of counselors reported lower stress levels during online counseling. No significant age or gender effects were found for digital stress. However, digital stress showed significant correlations with specific psychosomatic complaints (back pain, *r*=–0.26; *P*=.003; nervousness, *r*=–0.19; *P*=.03; sweat attacks, *r*=0.180; *P*=.04).

### EFA Results

Prior to the EFA, the items “documentation” and “costs for clients” were excluded due to content overlap with the item “time management,” and due to respondents showing an overall neutral response pattern (61% neutral). The other results of the EFA are reported in Table 1.

**Table 1.** Explorative factor analysis of the QueStrOn (Principal Axis, Varimax with Kaiser Normalization; N=174), with resulting 4-Factor Solution.

“insert suitable column head”		Factor			
		1	2	3	4
Factor 1: “Online Communication”					
Verbal expression of counselors		0.843	—^a^	—	—
Verbal expression of clients		0.747	—	—	—
Counseling goals		0.634	—	—	—
Factor 2: “Online Work Organization”					
Temporal flexibility		—	0.915	—	—
Perceived accessibility of counselors		—	0.553	—	—
Time management		—	0.491	—	—
Spatial flexibility		—	0.408	—	—
Client anonymity		—	0.395	—	—
Factor 3: “Online Work Content”					
Facial expressions and gestures		—	—	0.757	—
Management of crisis		—	—	0.598	—
Relationship building		0.410	—	0.501	—
Engagement of clients		—	0.401	0.488	—
Factor 4: “Online Framework”					
Existence of guidelines		—	—	—	0.519
Technical difficulties		—	—	0.431	0.449
Data privacy		0.402	—	—	0.446
Privacy of clients		—	—	—	0.353

a Factor loadings <0.30 are not depicted in this table.

The KMO value was 0.816, indicating sampling adequacy. The Bartlett test of sphericity was significant. All retained factors exceeded 0.4, and loadings below 0.3 were excluded. We found that a 4-factor solution best fit the collected data:

1. Factor 1, which was named “Online Communication,” includes items related to communication and goal attainment in online counseling.
2. Factor 2, which was named “Online Work Organization,” encompasses items related to flexibility, time management, and related organizational aspects.
3. Factor 3, named “Online Work Content,” covers items describing the counseling process and quality of the therapeutic relationship. The item “relationship building” exhibited cross-loadings on factor 1, likely due to its conceptual overlap with communication and work content. The item “engagement of clients” cross-loaded on factor 2. A possible explanation for this is that factor 2 includes client-centered questions. Although cross-loadings were noted, both items were retained and scored according to their higher loading on factor 3, as exclusion was not deemed necessary.
4. Factor 4, which was named “Online Framework,” reflects the general conditions of online counseling. Within factor 4, the item “data privacy” cross-loaded on factor 1, which can be explained by communication difficulties while protecting privacy. The item “technical difficulty” cross-loaded on factor 3, reflecting possible interruptions during therapeutic relationship building or crisis intervention. Nevertheless, both items remained within factor 4 due to their higher factor loadings.

### Reliability

To assess the reliability of the factors, internal consistency was evaluated by calculating Cronbach alpha for each factor, as well as item discriminatory power. The overall Cronbach α for the instrument was 0.870. The scales for “Online Work Organization,” “Online Work Content,” and “Online Communication” demonstrated good internal consistency, with Cronbach α values of 0.826 (Factor 1), 0.754 (Factor 2), and 0.745 (Factor 3), respectively. The factor 'Online Framework' though, showed low reliability with α=0.502.

### Regressions

To evaluate and confirm the predictive power of the QueStrOn, a predictive model was developed, which used “stress level in online counseling” as the dependent variable, while the 4 factors of the QueStrOn were included as predictors in a linear regression analysis (*R*²=0.271). The model was adjusted for age and gender. The ANOVA for the regression model was significant (*F*_5_=13.335; *P*<.001). Significant results were found for all 4 factors (Table 2). The regression results indicate that perceiving Online Work Organization, Online Communication, and the Online Framework as advantageous is associated with lower stress levels in online counseling compared to face-to-face counseling. Online Work Content also emerged as a significant predictor of stress level; however, its effect was in the opposite direction, indicating that higher demands in work content are associated with increased stress levels. Thus, a significant regression model was obtained, supporting the hypothesis that the QueStrOn explains variance in the personal stress levels of online counselors.

**Table 2.** Exploratory linear regression model between predictors (QueStrOn 4-factor solution) and health outcome (stress level in Online Counseling; N=174; *R*^2^=0.271).

EFA-factors	Standardized beta coefficient^a^	T^a^	*P* value^a^
Online Work Organization	0.274	3.527	<.001
Online Communication	0.274	3.428	<.001
Online Work Content	–0.172	–2.084	.04
Online Framework	0.274	3.663	<.001

a adjusted for age age and gender.

## Discussion

### Principal Findings

The main objective of the study was to develop and explore an instrument that identifies a specific risk profile for online counseling compared to face-to-face counseling. The initial model for factor analysis was based on preliminary interviews, expert opinions, and an extensive literature review and was pretested by online counselors. EFA then resulted in a 4-factor solution with 16 items, defined as (1) Online Work Organization, (2) Online Framework, (3) Online Work Content, and (4) Online Communication.

The factor “Online Work Organization” demonstrated good internal consistency. It primarily comprises counselor-centered questions (eg, feeling of constant availability) but also includes client-centered questions (eg, flexibility of clients). Previous studies have also found client flexibility in location and time alongside counselors’ sense of constant availability as significant factors influencing the relationship between work processes and adverse health effects [11,13,44-46].

The therapeutic relationship remains the core work content for achieving counseling goals. It can be subdivided into successful relationship building, effective crisis intervention, and sufficient client engagement [21,47,48]. Many studies have shown that these aspects foster a good therapeutic relationship and serve as milestones in the therapy process and progress [12,14,21,47,49]. All these content-related factors can be negatively impacted by deficits in verbal and nonverbal communication, which have been previously identified as pivotal influences [12,14,15].

The factor structure suggests 2 focal points for the introduction and development of online counseling formats. These include regulations to improve work organization, especially concerning time management and counselors’ accessibility; also, the promotion of therapeutic and method skills facilitates digital and online relationship building and maintenance. In addition, the factor structure implies that framework conditions related to digital transformation, such as data protection and privacy, which often play a more subordinate role in face-to-face contact, should receive focused attention in future online counseling research.

The subscale “Online Framework” exhibited only sufficient internal consistency. This could reflect the broad range of work and workplace design possibilities associated with digital work. Despite this result, the factor should not be dismissed, as it showed a strong predictive value for online counseling stress. While our previous interviews revealed associations and interactions between framework factors and other workplace health factors in digital contexts [11], few studies specifically examined organizational frameworks and their contribution to digital stress. Again, related aspects, such as data security and privacy, are frequently mentioned in the literature [1,13,22], underscoring the need for focused research on organizational framework conditions in digital social work.

The overall instrument showed strong internal consistency of α=0.870, with acceptable to satisfactory alphas across the 4 factors. These findings align well with theoretical assumptions and empirical findings.

To evaluate the instrument’s predictive utility for stress levels in online counselors, the factors were included in a linear regression model with “Online Stress” as the dependent variable. Online stress in our cohort correlated with psychosomatic complaints, suggesting an indirect link between QueStrOn factors and occupational health, though this finding warrants further investigation. The regression model explained approximately 27.1% of variance in stress levels. It is plausible that adding the demands associated with Online Work Organization increases perceived stress. While the model leaves a substantial proportion of variance unexplained, other nononline-specific stressors (eg, bureaucratic hurdles or client aggression) likely contribute substantially to the prediction of stress and should be integrated in future assessments [9,50]. Several existing instruments address these broader factors and should be considered in subsequent research to refine the understanding of online counseling stressors as outlined by the QueStrOn.

Interestingly, the factor “Online Work Content” showed an inverse relationship with stress levels. One possible explanation is that a poor therapeutic relationship may reduce counselors’ identification with the client’s problems, allowing a greater emotional distance and thus lower perceived stress compared to face-to-face counseling. It might also indicate that choosing a digital exchange induces less emotional commitment and reduces tendencies toward pathological altruism, a behavioral pattern often observed in social work [51]. Further research is needed to test this hypothesis.

The regression results underscore the importance of employer collaboration with counselors when implementing online counseling. Counselors’ perceptions of relationship management and work organization appear to influence their stress level. Occupational health literature suggests that elevated stress can impair health and increase absenteeism, highlighting the need for further research in this area.

Overall, the QueStrOn factors appear suitable for addressing additional challenges unique to online counseling. Future work will focus on refining the instrument to create a practicable, brief tool to assess online counseling stressors. This tool could supplement regular occupational risk assessments and help evaluate the impact of workplace interventions.

As this study, and the QueStrOn instrument, represent a first step in identifying key risk factors related to online counseling, several limitations should be acknowledged. The sample size was relatively small, so generalizability of results should be considered with caution. As the first instrument specifically targeting stressors in online counseling, direct comparisons regarding item effectiveness are not yet feasible. Therefore, the comparability of our explorative data is also limited. To date, much of the available information on the demands of online counseling is qualitative. In this context, our findings align well with pre-existing conceptions of stressors in online counseling.

In addition, participants primarily conducted face-to-face counseling with online counseling as an adjunct to their daily working routine. Consequently, results may be influenced by the dual-method work environment and could evolve with increasing experience and technological advances. Counselors primarily trained in digital methods might experience fewer challenges and health effects. Since most counselors use both asynchronous and synchronous formats similarly, the lines between digital and face-to-face stressors might be blurred. Furthermore, this study was conducted in the first year of the COVID-19 pandemic, a period marked by exceptional stress due to isolation, lockdowns, health uncertainties, and information overload. These extraordinary circumstances likely influenced consultants’ responses, given the parallel heightened demand for counseling services and the rapid necessity for digital or online delivery. Further and longitudinal research in more stable social conditions should answer the question of whether the pandemic led to a situational bias in our study.

### Conclusion

This study presents the development and validation of QueStrOn, a novel instrument designed to assess stressors specific to online counseling. Based on the Job Demands-Resources model, 4 key factors were identified, including Online Work Organization, Online Communication, Online Work Content, and Online Framework. These dimensions significantly predicted perceived stress levels, with challenges in communication and therapeutic engagement associated with increased stress, while positive evaluations of organizational and structural conditions correlated with reduced stress.

The findings highlight the need for targeted occupational health strategies addressing the unique demands of online counseling and, prospectively, any online-based interactive task between humans. QueStrOn offers a reliable and theory-driven tool to support systematic risk assessment and inform future interventions aimed at improving counselor well-being in digital service delivery contexts.

## Abbreviations

BZgA: Bundeszentrale für gesundheitliche Aufklärung
EFA: exploratory factor analysis
GDA: Gemeinsame Deutsche Arbeitsschutzstrategie
KMO: Kaiser-Meyer-Olkin
QueStrOn: Questionnaire to Assess Stressors in Online Counseling
